# Keap1在非小细胞肺癌中的表达及与化疗疗效相关性的研究

**DOI:** 10.3779/j.issn.1009-3419.2012.10.05

**Published:** 2012-10-20

**Authors:** 宝山 曹, 翔 朱, 森 陈, 宇 肖, 莉 梁

**Affiliations:** 1 100191 北京，北京大学第三医院肿瘤治疗中心化疗与放射病科 Department of Oncology and Radiation Sickness, Cancer Center, Beijing 100191, China; 2 100191 北京，北京大学第三医院肿瘤治疗中心病理科 Department of Pathology, Peking University Third Hospital, Beijing 100191, China

**Keywords:** 肺肿瘤, Keap1, 化疗, 无进展生存期, Lung neoplasms, Kelch-like ECH-associated protein 1, Chemotherapy, Progression free survival

## Abstract

**背景与目的:**

已有研究表明Kelch样环氧氯丙烷相关蛋白1（Kelch-like ECH-associated protein1, Keap1）与铂类耐药相关。本研究旨在探讨Keap1在进展期非小细胞肺癌（non-small cell lung cancer, NSCLC）中的表达及其与一线含铂化疗方案疗效的相关性。

**方法:**

应用免疫组化检测50例进展期NSCLC患者组织标本中Keap1的表达。

**结果:**

进展期NSCLC患者中Keap1高表达率为26.0%；Keap1表达水平与化疗疗效、无进展生存期（progression free survival, PFS）有关（*P* < 0.05），而与性别、年龄、吸烟、病理类型、分化程度、转移和总生存期无关（*P* > 0.05）。Keap1高表达组的中位PFS明显高于低表达组（*P*=0.002）。多因素分析表明Keap1表达水平是一线化疗方案PFS的独立预测因素（*P*=0.007）。

**结论:**

Keap1与进展期NSCLC一线化疗疗效和PFS有关，Keap1可能成为新的化疗疗效预测指标。

## Abstract (en)

**Background and objective:**

It has been proven that Kelch-like ECH-associated protein 1 (Keap1) expression was correlated with the chemoresistance of platinum drugs. The aim of this study is to investigate the protein expression levels of Keap1 in non-small cell lung cancer (NSCLC) patients as well as to correlate its expression with the response rate (RR), progression-free survival (PFS), and overall survival (OS) of patients treated with platinum-based first-line chemotherapy.

**Methods:**

The immunohistochemical analysis of Keap1 expression was performed using tumor samples from 50 patients with stage Ⅲ or Ⅳ NSCLC.

**Results:**

The high expression ratio of Keap1 was 26.0%. The Keap1 expression was significantly correlated with the RR and PFS after platinum-based chemotherapy (*P* < 0.05) but not with the gender, age, smoking, pathology type, differentiation, metastasis, and OS (*P* > 0.05). The PFS was significantly longer in patients with high Keap1 expression than in those with low/negative expression (*P*=0.002). Furthermore, the level of Keap1 expression was an independent predictive factor of PFS after platinum-based first-line chemotherapy (*P*=0.007).

**Conclusion:**

The expression of Keap1 was significantly correlated with the RR and PFS of platinum-based first-line chemotherapy. Therefore, Keap1 may be a useful biomarker for predicting the chemosensitivity of patients with advanced-stage NSCLC.

目前肺癌死亡率已位居男、女性恶性肿瘤的第一位^[1]^，全球每年至少有160万的新发病例和130万的死亡病例。非小细胞肺癌（non-small cell lung cancer, NSCLC）占肺癌的80%左右^[2]^，发现时多处于进展期，化疗仍是进展期NSCLC治疗的主要手段。含铂两药方案是一线化疗首选方案，耐药是化疗失败的主要原因。目前关于肿瘤耐药机制尚不完全清楚，因此探索与肿瘤耐药相关的生物指标，对提高化疗疗效和患者生活质量显得尤为重要。

Kelch样环氧氯丙烷相关蛋白-1（Kelch-like ECH-associated protein1, Keap1）是细胞应对氧化应激和亲电性应激损伤的重要防御基因之一^[3]^。基础研究^[3, 4]^表明Keap1的表达水平与NSCLC细胞对卡铂、顺铂、依托泊苷等药物的敏感性相关。因此，Keap1表达水平或许与NSCLC患者含铂化疗方案疗效相关。为此，本研究通过免疫组化方法检测了50例进展期NSCLC患者组织标本中Keap1的表达水平，并分析了Keap1表达水平与患者临床特征和一线含铂化疗方案疗效之间的关系。

## 1 对象与方法

### 1.1 研究对象

选取2008年1月-2011年12月在北京大学第三医院接受化疗的共50例NSCLC患者。入组前需病理确诊为NSCLC；不能手术切除的Ⅲ期和Ⅳ期患者（依据国际肺癌研究协会颁布的第7版分期标准^[5]^）；有足够的病理组织标本供免疫组化检测；一线方案是含铂两药方案；治疗前、治疗2个周期和4个周期后均有影像学检查（胸腹部CT、头颅MRI）。共纳入符合条件的病例50例，包括男性29例，女性21例；年龄范围48岁-77岁，中位年龄64岁；鳞癌患者23例，腺癌患者27例；Ⅲ期患者20例，Ⅳ期患者30例；接受紫杉类药物联合铂类方案化疗患者10例，接受吉西他滨联合铂类方案化疗患者28例，接受长春瑞宾联合铂类化疗患者5例，接受培美曲塞联合铂类化疗患者4例，其它药物联合铂类化疗患者3例。

### 1.2 随访及后续治疗

所有患者每3个月通过定期来院或电话随访，随访开始时间为2008年1月，末次随访时间为2012年3月1日，最短随访时间为3个月，最长为50个月。50例患者中仅接受1种治疗方案的患者14例，接受≥2种治疗方案的患者36例；后续治疗中接受放疗患者5例，根治性手术切除2例，接受表皮生长因子受体酪氨酸激酶抑制剂（epidermal growth factor receptor tyrosine kinase inhibitor, EGFR-TKI）靶向治疗患者13例。

### 1.3 临床资料收集及疗效评价标准

记录患者临床特征、化疗方案和影像学指标。疗效指标包括近期和远期疗效。近期疗效按照实体瘤疗效评价标准1.1版^[6]^分为完全缓解（complete response, CR）、部分缓解（partial response, PR）、疾病稳定（stable disease, SD）和疾病进展（progressive disease, PD），获得CR或PR的患者4周或以后确认。远期疗效为无疾病进展生存期（progression free survival, PFS）和总生存期（overall survival, OS）。PFS定义为从治疗开始至疾病进展或任何原因导致死亡的时间，OS定义为从初次治疗开始至死亡或随访终点时间。

### 1.4 免疫组化检测Keap1表达

#### 1.4.1 实验方法

活检组织标本经10%甲醛固定后，常规石蜡包埋，切片，4 μm厚度。采用免疫组化SP法检测（兔抗人Keap1抗体购于proteintech^®^公司，免疫组化二抗SP检测试剂盒购于北京中杉金桥生物技术有限公司，Keap1抗体按1:100稀释），应用PBS代替一抗作为阴性对照，按照试剂说明书进行操作。

#### 1.4.2 结果判定

采用双盲法，由两位独立的病理医师进行阅片，Keap1抗原阳性反应位于细胞浆内。随机选取5个高倍镜视野（200倍），每个视野记数200个肿瘤细胞。共计数1, 000个细胞。阳性结果参照Solis等研究^[7]^的评判标准，按染色强度分为：0分，无染色；1分，染色呈淡黄色；2分，染色呈棕黄色；3分，染色呈棕褐色；按阳性细胞比例分为0%-100%；按照染色强度和阳性细胞比例乘积判定免疫组织化学表达水平。Keap1低或不高表达是指二者乘积 < 150%，反之为高表达。

### 1.5 统计学方法

应用SPSS 17.0统计学软件分析。率的比较采用卡方检验或*Fisher*精确检验，相关性检验采用*Pearson*检验，多因素分析采用*Logistic*回归模型（逐步后退法）。*Kaplan-Meier*方法进行生存分析，*Log-rank*检验差异性，多因素分析采用*Cox*多因素分析模型。*P* < 0.05为差异有统计学意义。

## 2 结果

### 2.1 随访及疗效

随访率为100%。中位随访时间16个月，随访范围3个月-50个月。近期疗效中无患者为CR，17例（34.0%）患者为PR，23例（46.0%）患者为SD，10例（20.0%）患者为PD。因3例获得PR患者中2例接受了根治性手术切除，1例接受了根治性放射治疗，后期检查方式和间隔差别较大，故在评价远期疗效时将其剔除。远期疗效：PFS为1个月-12个月，中位PFS为5个月；OS为3个月-50个月，中位OS为16个月。

### 2.2 Keap1蛋白检测结果及与临床特征间的关系

Keap1主要表达在细胞浆中（图 1）。Keap1高表达率为26.0%（13/50）。Keap1在PR患者中的高表达率为47.1%（8/17），明显高于PD患者的10.0%（1/10）（*P*=0.047）。但Keap1表达水平在性别、年龄、病理类型、分化程度、远处转移、吸烟和化疗方案组间无差异（*P*＞0.05），见表 1。

**1:**
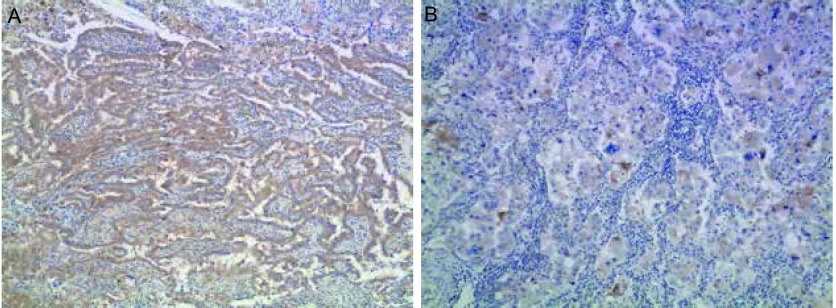
免疫组化检测Keap1在肺癌中的表达情况（×100）。A：Keap1高表达；B：Keap1低表达 Expression of Keap1 in lung cancer (×100). A: Keap1 high expression; B: Keap1 low expression

**1:** Keap1表达水平同接受化疗的NSCLC患者临床特征关系（*n*=50） The relationship between the protein expression level of Keap1 and clinical features of NSCLC patients with chemotherapy (*n*=50)

Clinical characterstic	*n*	High expression of Keap1	*χ*^2^	*P*
Gender			2.582	0.108
Male	29	10 (34.5%)		
Female	21	3 (14.3%)		
Age (yr)			0.008	0.928
< 70	38	10 (26.3%)		
≥70	12	3 (25.0%)		
Pathological type			< 0.001	0.990
SQC	23	6 (26.1%)		
ADC	27	7 (25.9%)		
Differentiation			3.175	0.204
Low	2	1 (50.0%)		
Moderate	33	6 (18.2%)		
High	15	6 (40.0%)		
Smoking history			1.661	0.198
Yes	31	10 (32.3%)		
No	19	3 (15.8%)		
Metastasis			0.017	0.895
M0	20	5 (25.0%)		
M1	30	8 (26.7%)		
Chemotherapeutic regimen			2.022	0.732
Taxanes-based	10	3 (30.0%)		
Gemcitabine-based	28	8 (28.6%)		
Novibine-based	5	0		
Pemtrexed-based	4	1 (25.0%)		
Others	3	1 (33.3%)		
Response			6.135	0.047
PR	17	8 (47.1%)		
SD	23	4 (17.4%)		
PD	10	1 (10.0%)		
SQC: squamous cell carcinoma; ADC: adenocarcinoma; NSCLC: non-small cell lung cancer; PR: partial response; SD: stable disease; PD: progressive disease.				

### 2.3 Keap1表达与化疗疗效、PFS、OS相关性分析

*Pearson*相关分析表明，Keap1表达与化疗疗效（*r*=-0.327, *P*=0.020）和PFS（*r*=0.439, *P*=0.002）相关，但与OS（*r*=0.018, *P*=0.904）无关，Keap1高表达NSCLC患者的疗效及PFS明显优于低或不表达者。

### 2.4 生存分析

*Kaplan-Meier*生存分析显示Keap1高表达NSCLC患者组的中位PFS和OS分别为8.0个月和19.0个月；低表达组分别为4.0个月和19.5个月，Keap1高表达组中位PFS明显高于低表达组（*P*=0.002，图 2A），但两组在OS方面无统计学差异（*P*=0.760，图 2B）。

**2:**
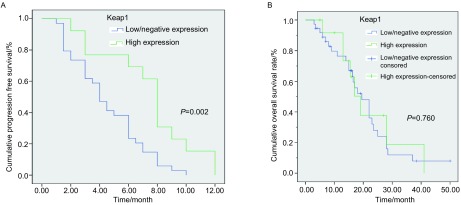
*Kaplan-Meier*生存曲线分析。A：Keap1高表达和低/不表达NSCLC患者的PFS生存曲线；B：Keap1高表达和低/不表达NSCLC患者的OS生存曲线 *Kaplan-Meier* cumulative survival time curves analysis. A: The PFS curves of Keap1 high expression and low/negative expression group of NSCLC patients; B: The OS curves of Keap1 high expression and low/negative expression group of NSCLC patients. PFS: progression free survival

### 2.5 多因素分析

校正性别、年龄、病理类型、转移等因素后，*Logistic*回归分析表明Keap1表达水平有成为一线化疗疗效的独立预测指标的趋势（*P*=0.065），见表 2。校正性别、年龄、病理类型、分化程度、化疗方案、转移、接受化疗方案种类、接受EGFR-TKI治疗与否等因素后，多因素分析表明Keap1表达水平是一线化疗PFS的独立预测因素（*P*=0.007），但不是OS的独立预测因素（*P*=0.700）。病理类型（*P*=0.026）、后续是否接受EGFR-TKI治疗（*P*=0.007）及接受化疗方案种类（*P*=0.046）是OS的预测因素，见表 3。

**2:** 回归模型比较一线化疗疗效相关的各组变量（*n*=50） Comparsion of variable for chemotherapy induced response (*n*=50)

Characteristic	Regression coefficient *β*	Standard error	Wald	*P*	Exp(B)	95%CI
Gender	0.481	0.910	0.279	0.597	1.617	0.272-9.615
Age	-0.569	1.285	0.196	0.658	0.566	0.046-7.030
Pathological type	0.617	0.959	0.414	0.520	1.853	0.283-12.147
Metastasis	0.442	0.973	0.206	0.650	1.555	0.231-10.471
Keap1	-2.204	1.194	3.407	0.065	0.110	0.011-1.146

**3:** 多因素分析NSCLC特异性生存率的预后因素（*n*=47） *Cox* regression analysis of the disease-specific survival of NSCLC patients (*n*=47)

Characteristic	Regression coefficient *β*	Standard error	Wald	*P*	Exp(B)	95%CI
Progression free survival						
Gender	0.339	0.478	0.503	0.478	1.403	0.550-3.577
Age	0.418	0.433	0.929	0.335	1.519	0.649-3.551
Pathological type	0.186	0.380	0.240	0.624	1.205	0.572-2.538
Differentiation	-0.131	0.316	0.173	0.677	0.877	0.472-1.628
Metastasis	-0.023	0.427	0.003	0.956	0.977	0.423-2.254
Chemotherapeutic regimen	0.237	0.221	1.151	0.283	1.268	0.822-1.956
Keap1	-1.079	0.398	7.358	0.007	0.340	0.156-0.741
Overall survival						
Gender	0.288	0.657	0.193	0.661	1.334	0.368-4.831
Age	-0.421	0.683	0.380	0.538	0.657	0.172-2.504
Pathological type	-2.212	0.992	4.976	0.026	0.109	0.016-0.765
Differentiation	0.863	0.587	2.164	0.141	2.370	0.751-7.486
Number of chemo therapeutic regimen	-0.904	0.454	3.966	0.046	0.405	0.166-0.986
EGFR-TKI treatment	-2.375	0.880	7.290	0.007	0.093	0.017-0.522
Keap1	-0.276	0.716	0.149	0.700	0.759	0.186-3.088
EGFR-TKI: epidermal growth factor receptor tyrosine kinase inhibitor.						

## 3 讨论

Keap1是细胞防御氧化和亲电性应激损伤的重要蛋白，其与核因子E2相关因子2（nuclear factor erythroid-2-related factor 2, Nrf2）组成重要的细胞防御体系。生理状况下，Keap1结合Nrf2，并与E3泛素化连接酶结合，通过泛素化介导Nrf2蛋白降解，维持细胞浆内Nrf2处于较低水平。一旦细胞受到外源性或内源性的氧化应激或亲电性应激刺激，Keap1便成为敏感的传感器，其通过对自身半胱氨酸残基的修饰，阻止Nrf2降解，并促进Nrf2释放，累积的Nrf2进入细胞核内，激活抗氧化反应元件（antioxidant response element, ARE），进而激活ARE下游的细胞保护基因，包括：①细胞内氧化还原基因，如谷氨酸半胱氨酸连接酶、血红素氧合酶-1等；②Ⅱ相解毒基因，如谷胱甘肽-S转移酶、NAD（P）H苯醌氧化还原酶-1等；③编码转运蛋白的基因，如多药耐药蛋白（multidrug resistant protein, MRP）等，从而保护细胞免受氧化应激或亲电性应激所致损伤^[3, 8]^。

Solis等^[7]^应用免疫组化的方法发现Keap1高表达组NSCLC患者的PFS和OS明显高于低表达组；Takahashi等^[9]^和Li等^[10]^应用基因检测方法证实NSCLC Keap1的失活与预后差密切相关。近期研究^[3, 11-13]^表明Keap表达下降后Nrf2表达升高，导致肺癌细胞对阿霉素、足叶乙甙、顺铂和紫杉类药物耐药。因此Keap1表达水平除与NSCLC患者预后相关，还应与化疗疗效相关。但目前缺乏Keap1表达水平与进展期NSCLC患者一线化疗疗效的相关研究。因进展期患者组织标本通常是通过支气管镜或CT穿刺活检获得，组织标本较小，而免疫组化方法所需组织标本量明显少于基因检测方法。因此，本研究以临床肺穿刺或支气管活检标本为研究对象，应用免疫组织化学方法检测Keap1蛋白，拟明确进展期NSCLC患者中Keap1表达情况及与一线化疗疗效的相关性。

本研究结果显示进展期NSCLC患者中Keap1表达存在个体差异，Keap1高表达率为26.0%。Keap1表达产生个体差异机制包括：①遗传背景本身所致；②Keap1自身突变不同所致，近期多项研究发现NSCLC标本中检测出Keap1突变^[10, 14, 15]^，突变导致Keap1表达下降或缺失；③Keap1甲基化所致，Wang等^[11]^和Muscarella等^[16]^发现NSCLC组织标本和细胞株中存在Keap1基因启动子CpG岛甲基化，甲基化导致Keap1功能减低；④*Nrf2*突变所致，*Nrf2*突变的NSCLC细胞株中Nrf2过表达，间接抵消Keap1功能^[17]^；⑤外源性刺激Nrf2活性增强，间接减弱Keap1功能所致，张凯茹等^[18]^发现耐顺铂的A549细胞株中Nrf2水平较亲本的A549明显升高。上述因素可以单一或同时存在。

本研究中Keap1高表达的NSCLC患者组获得PR率高于低表达或不表达患者组。其原因可能为在Keap1不表达或低表达的患者中，Keap1对Nrf2调控受限，从而促使Nrf2进入细胞核内激活ARE的下游基因，如Ⅱ相解毒基因、编码转运蛋白的基因等，从而促进药物解毒、外排，产生耐药^[3]^；而Keap1高表达者，可维持Nrf2处于较低水平，进而提高药物敏感性^[4]^。结合本研究结果，Keap1表达水平或许是预测化疗疗效的理想指标。

本研究中Keap1高表达组患者的PFS明显延长，可能与Keap1高表达患者组客观缓解率高或Keap1高表达的肿瘤细胞增殖、转移能力下降^[4, 19]^相关。但在OS方面，本研究发现Keap1表达水平与OS无关，与Solis^[7]^和Merikallio^[20]^研究结果不同。其差异产生的原因可能在于：①Keap1表达情况随着外界环境刺激发生改变；②后续治疗不平衡，如患者接受化疗方案的种类以及是否接受EGFR-TKI治疗等不同；③二者研究对象不同，本研究涉及均是进展期患者，而Solis^[7]^和Merikallio^[20]^研究中多为术后患者。

本研究的主要限制在于样本量偏小，可能存在选择偏移。但本研究发现Keap1表达水平与一线化疗疗效、PFS相关，Keap1是PFS的独立预测因素。因此Keap1或许可成为预测NSCLC化疗疗效的生物指标，需要进一步扩大样本量，进行前瞻性研究验证Keap1对化疗疗效预测的临床价值。

## References

[1] Jemal A, Bray F, Center MM (2011). Global Cancer Statistics. CA Cancer J Clin.

[2] Mountain CF (1997). Revisions in the international system for staging lung cancer. Chest.

[3] Singh A, Misra V, Thimmulappa RK (2006). Dysfunctional KEAP1-NRF2 interaction in non-small-cell lung cancer. PLoS Med.

[4] Qu LY, Gao P, Wang HY (2010). Nrf2 down-regulated cell line H460-N5 with Keap1 over-expression increased sensitivity to anti-cancer drugs. Zhejiang Da Xue Xue Bao Yi Xue Ban.

[5] Detterbeck FC, Boffa DJ, Tanoue LT (2009). The new lung cancer staging system. Chest.

[6] Eisenhauer EA, Therasse P, Bogaerts J (2009). New response evaluation criteria in solid tumours: revised RECIST guideline (version 1.1). Eur J Cancer.

[7] Solis LM, Behrens C, Dong W (2010). Nrf2 and Keap1 abnormalities in non-small cell lung carcinoma and association with clinicopathologic features. Clin Cancer Res.

[8] Que LL, Wang HX, Cao BS (2011). The regulation and functions of transcription factor Nrf2 in cancer chemoprevention and chemoresistance. J Chin Pharm Sci.

[9] Takahashi T, Sonobe M, Menju T (2010). Mutations in Keap1 are a potential prognostic factor in resected non-small cell lung cancer. J Surg Oncol.

[10] Li QK, Singh A, Biswal S (2011). *KEAP1* gene mutations and NRF2 activation are common in pulmonary papillary adenocarcinoma. J Hum Genet.

[11] Wang R, An J, Ji F (2008). Hypermethylation of the *Keap1* gene in human lung cancer cell lines and lung cancer tissues. Biochem Biophys Res Commun.

[12] Ramos-Gomez M, Kwak MK, Dolan PM (2001). Sensitivity to carcinogenesis is increased and chemoprotective efficacy of enzyme inducers is lost in nrf2 transcription factor-deficient mice. Proc Natl Acad Sci USA.

[13] Shim GS, Manandhar S, Shin DH (2009). Acquisition of doxorubicin resistance in ovarian carcinoma cells accompanies activation of the NRF2 pathway. Free Radic Biol Med.

[14] Ohta T, Iijima K, Miyamoto M (2008). Loss of Keap1 function activates Nrf2 and provides advantages for lung cancer cell growth. Cancer Res.

[15] Yoo NJ, Kim HR, Kim YR (2012). Somatic mutations of the *KEAP1* gene in common solid cancers. Histopathology.

[16] Muscarella LA, Parrella P, D'Alessandro V (2011). Frequent epigenetics inactivation of *KEAP1* gene in non-small cell lung cancer. Epigenetics.

[17] Shibata T, Saito S, Kokubu A (2010). Global downstream pathway analysis reveals a dependence of oncogenic NF-E2-related factor 2 mutation on the mTOR growth signaling pathway. Cancer Res.

[18] Zhang KR, Yan HQ, Wang Y (2009). Expression and significance of nrf2 and its target genes in pulmonary adenocarcinoma A549 cells resistant to Cisplatin. Zhongguo Fei Ai Za Zhi.

[19] Singh A, Boldin-Adamsky S, Thimmulappa RK (2008). RNAi-mediated silencing of nuclear factor erythroid-2-related factor 2 gene expression in non-small cell lung cancer inhibits tumor growth and increases efficacy of chemotherapy. Cancer Res.

[20] Merikallio H, Paakko P, Kinnula VL (2012). Nuclear factor erythroid-derived 2-like 2 (Nrf2) and DJ1 are prognostic factors in lung cancer. Hum Pathol.

